# Supplementary material for: Development of a competition assay to assess the in vitro fitness of dengue virus serotypes using an optimized serotype-specific qRT-PCR
Source: PLoS One. 2025 Dec 15;20(12):e0339033. doi: 10.1371/journal.pone.0339033 (PMC12704846; doi:10.1371/journal.pone.0339033)
Supplement: S1 File — (DOCX) [file pone.0339033.s001.docx]

**Sequence of the DENV RNA calibrator “iv-RNA 4”:**

gaattcgcccttatccatgcccatcaccaatggatgacaacagaagacatgttgtcagtgtggaatagggtttggatagaggaaaacccatggatggaggataaaacccatatatccagttgggaagatgttccatacttaggaaaaagggaagatcagtggtgtggatccctgataggcttaacagcaagggccacctgggccactaatatacaagtggccataaaccaagtgagaaggcttattgggaatgagaattatctagattacatgacatcaatgaagagattcaagaatgagagtgatcccgaaggggcactctggtaagtcaacacattcacaaaacaaaggaaaataagaaatcaaacaaggcaagaagtcaggccggattaagccatagtacggtaagagctatgctgcctgtgagccccgtctaaggacgtaaaatgaagtcaggccggaagccacggtttgagcaaaccgtgctgcctgtagctccatcgtggggatgtaaaaacccgggaggctgcaacccatggaagctgtacgcatggggtagcagactagtggttagaggagacccctcccaaaacacaacgcagcagcggggcccaacaccaggggaagctgtaccctggtggtaaggactagaggttagaggagaccccccgcataacaataaacagcatattgacgctgggagagaccagagatcctgctgtctctacagcatcattccaggcacagaacgccagaaaatggaatgaagggcgaattc
