# Supplementary material for: Development of a competition assay to assess the in vitro fitness of dengue virus serotypes using an optimized serotype-specific qRT-PCR
Source: PLoS One. 2025 Dec 15;20(12):e0339033. doi: 10.1371/journal.pone.0339033 (PMC12704846; doi:10.1371/journal.pone.0339033)
Supplement: S1 Table — (DOCX) [file pone.0339033.s002.docx]

**Supplementary table 1. GenBank references of sequences used to design primers and probes of the optimized serotype-specific qRT-PCR.**

| Strains DENV-3 | GenBank Assession number | GenBank definition |
| --- | --- | --- |
| NC 001475.2 | [NC_001475](https://www.ncbi.nlm.nih.gov/nuccore/163644368) | Dengue virus 3, complete genome NCBI Reference Sequence: NC_001475.2 |
| Malaysia CNR 17046/2012 | [MF004386](https://www.ncbi.nlm.nih.gov/nuccore/1377039228) | Dengue virus type 3 isolate Malaysia_CNR_17046/2012 polyprotein gene, complete cds |
| 080606 | [KY794790](https://www.ncbi.nlm.nih.gov/nuccore/1343894117) | Dengue virus type 3 isolate 080606, complete genome |
| MS12007786 | [MK005258](https://www.ncbi.nlm.nih.gov/nuccore/MK005258.1) | Dengue virus type 3 isolate MS12007786 |
| PF91/090891-22130 | [JQ920476](https://www.ncbi.nlm.nih.gov/nuccore/JQ920476.1) | Dengue virus 3 isolate PF91/090891-22130 polyprotein gene, complete cds |
| WF95/050495-1650 | [JQ920488](https://www.ncbi.nlm.nih.gov/nuccore/JQ920488.1) | Dengue virus 3 isolate WF95/050495-1650 polyprotein gene, complete cds |
| NC89/060289-283 | [JQ920481](https://www.ncbi.nlm.nih.gov/nuccore/JQ920481.1) | Dengue virus 3 isolate NC89/060289-283 polyprotein gene, complete cds |
| SLEMAN-1280-AC25 | [MW946955](https://www.ncbi.nlm.nih.gov/nuccore/2027261380) | Dengue virus type 3 isolate SLEMAN-1280-AC25 |
| MD 33 R4 DENV3 III India | [MN253125](https://www.ncbi.nlm.nih.gov/nuccore/1739648312) | Dengue virus type 3 isolate MD_33_R4_DENV3_III_India |
| DENV-3/VN/BID-V1903/2008 | [KF955459](https://www.ncbi.nlm.nih.gov/nuccore/573974397) | Dengue virus type 3 isolate DENV-3/VN/BID-V1903/2008 |
