# Supplementary figures and images for: Development of a competition assay to assess the in vitro fitness of dengue virus serotypes using an optimized serotype-specific qRT-PCR
Source: PLoS One. 2025 Dec 15;20(12):e0339033. doi: 10.1371/journal.pone.0339033 (PMC12704846; doi:10.1371/journal.pone.0339033)

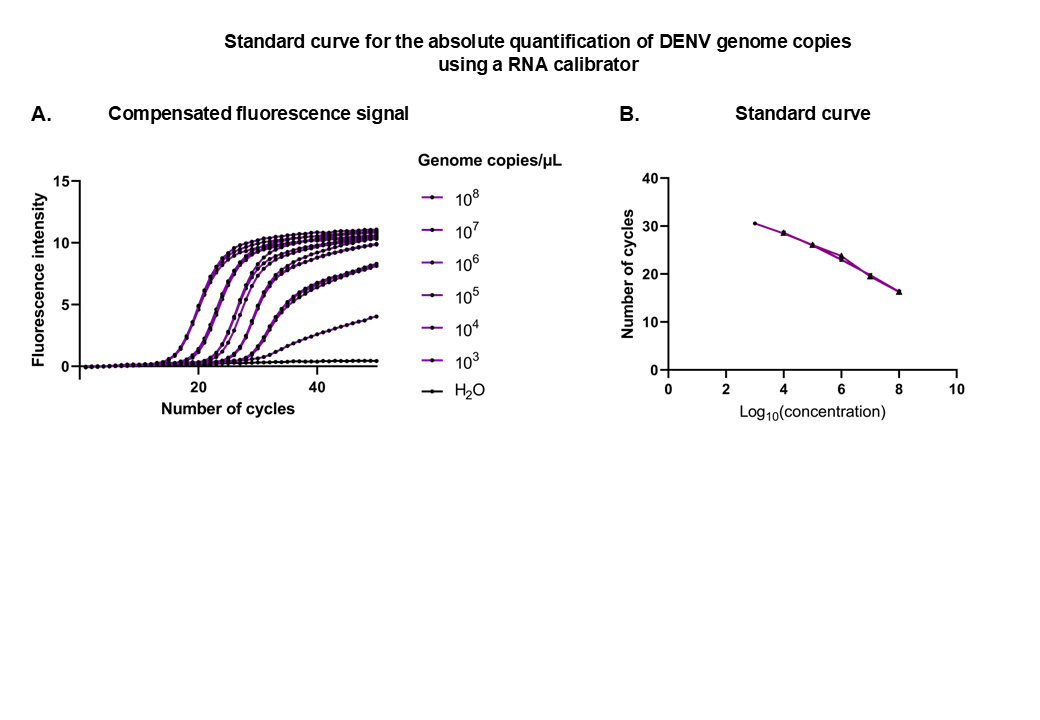

Supplement: S1 Fig — A. Compensated fluorescence intensity as a function of the number of PCR cycles for each dilution of the RNA calibrator. A signal was detected for the dilutions 108 to 103 copies/µL of the RNA calibrator. The limit of detection of this qRT-PCR was thus 103 copies/µL. B. Number of PCR cycles as a function of the logarithmic transform of the concentration of the RNA calibrator. Technical duplicates were run simultaneously. (TIF) [file pone.0339033.s003.tif]

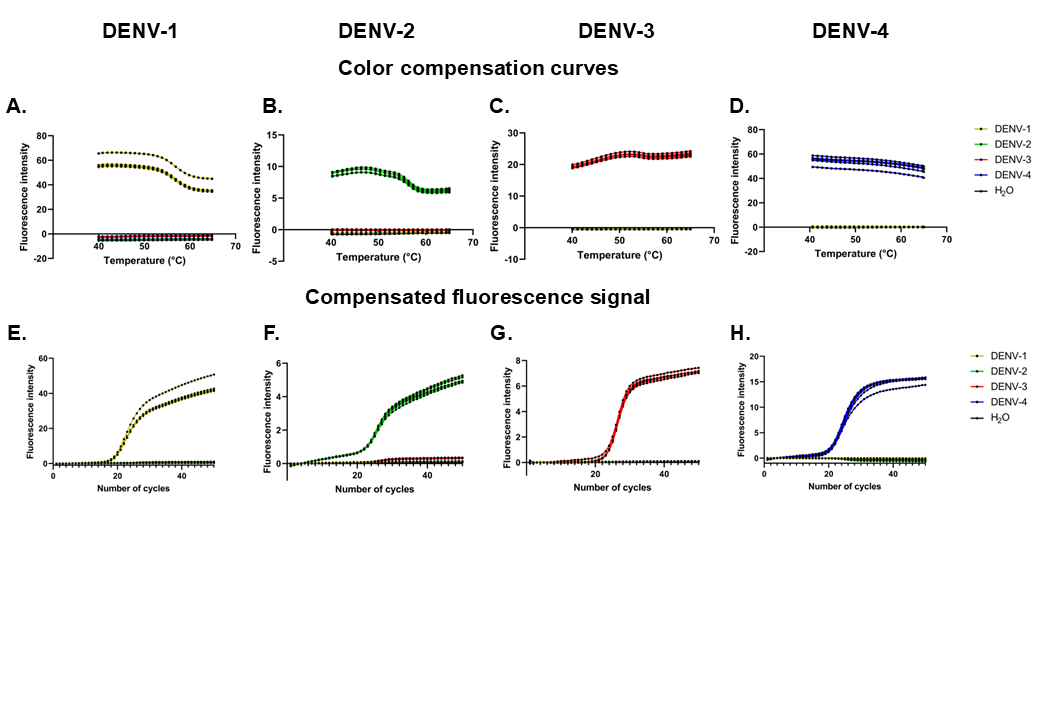

Supplement: S2 Fig — A-D. Color compensation curves for DENV-1 (A.), DENV-2 (B.), DENV-3 (C.) and DENV-4 (D.). E.-H. Fluorescence signal intensity as a function of the number of PCR cycles after color compensation for DENV-1 (E.), DENV-2 (F.), DENV-3 (G.) and DENV-4 (H.). (TIF) [file pone.0339033.s004.tif]

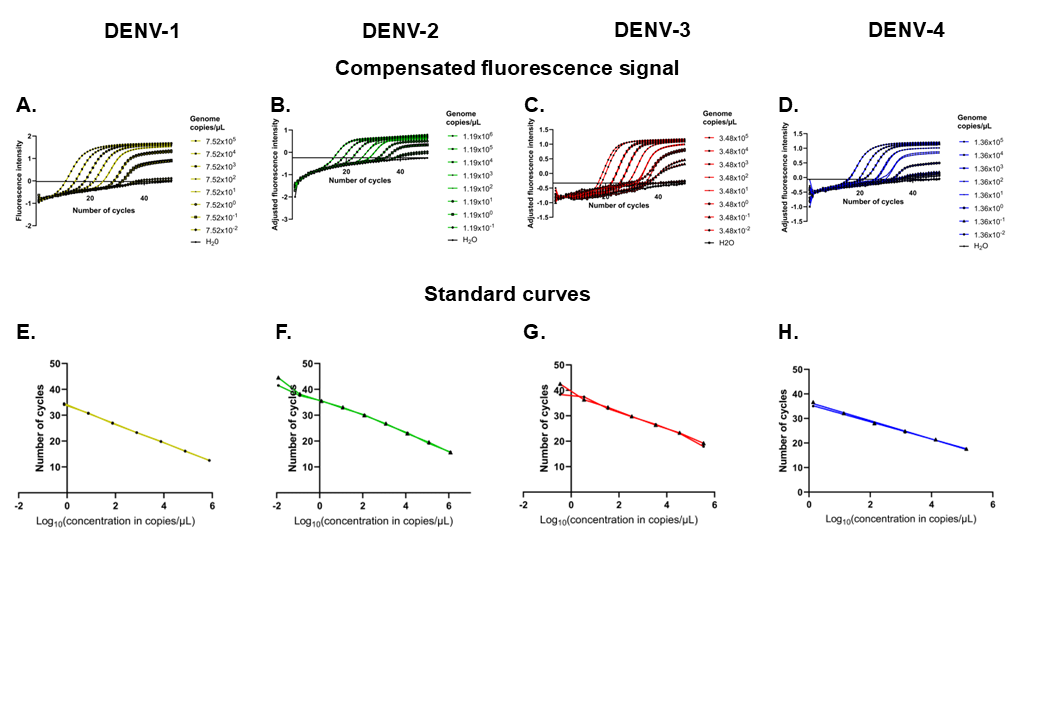

Supplement: S3 Fig — A-D. Fluorescence signal intensity as a function of the number of PCR cycles after color compensation and manual threshold adjustment on the negative control H2O for DENV-1 (A.), DENV-2 (B.), DENV-3 (C.) and DENV-4 (D.). Intersect of the X axis on the Y axis has been set at the threshold adjusted on the negative control. F-H. Standard curves for DENV-1 (E.), DENV-2 (F.), DENV-3 (G.) and DENV-4 (H.). (TIF) [file pone.0339033.s005.tif]

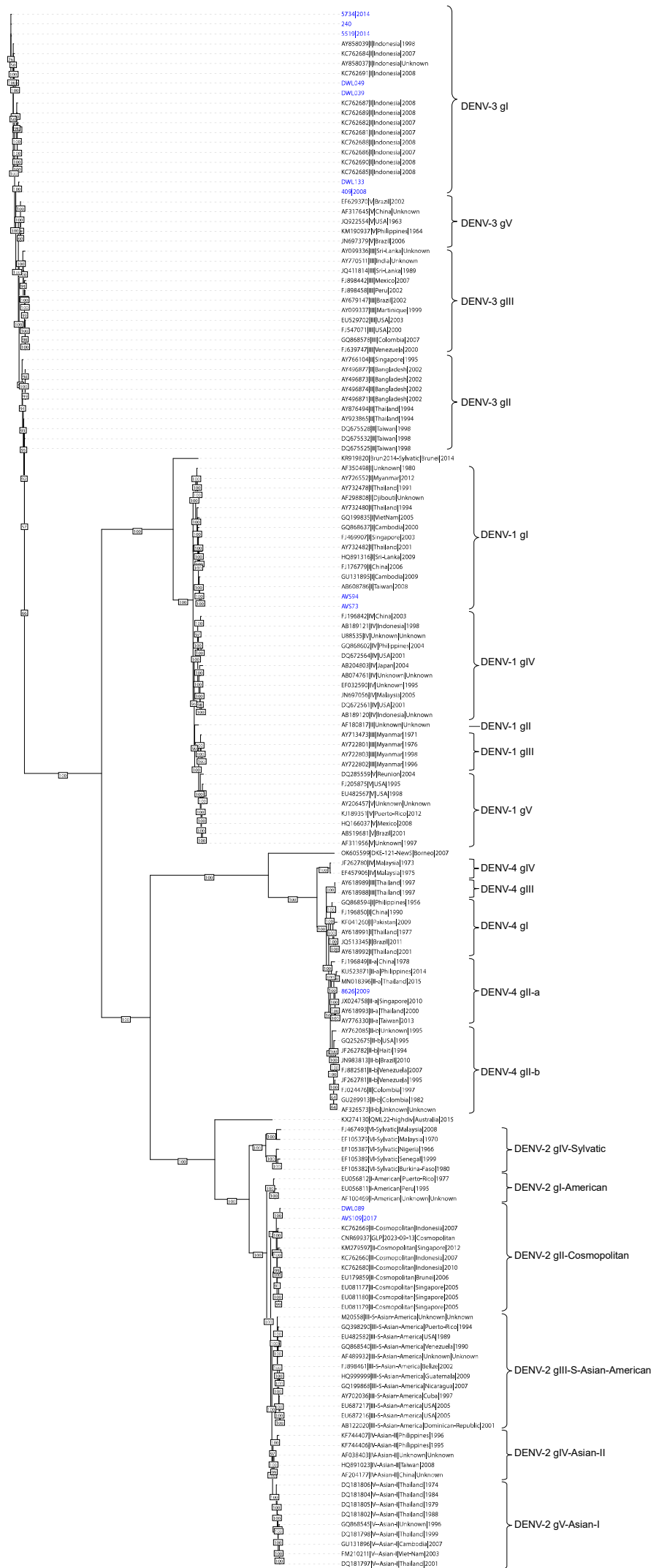

Supplement: S4 Fig — (PDF) [file pone.0339033.s006.pdf]

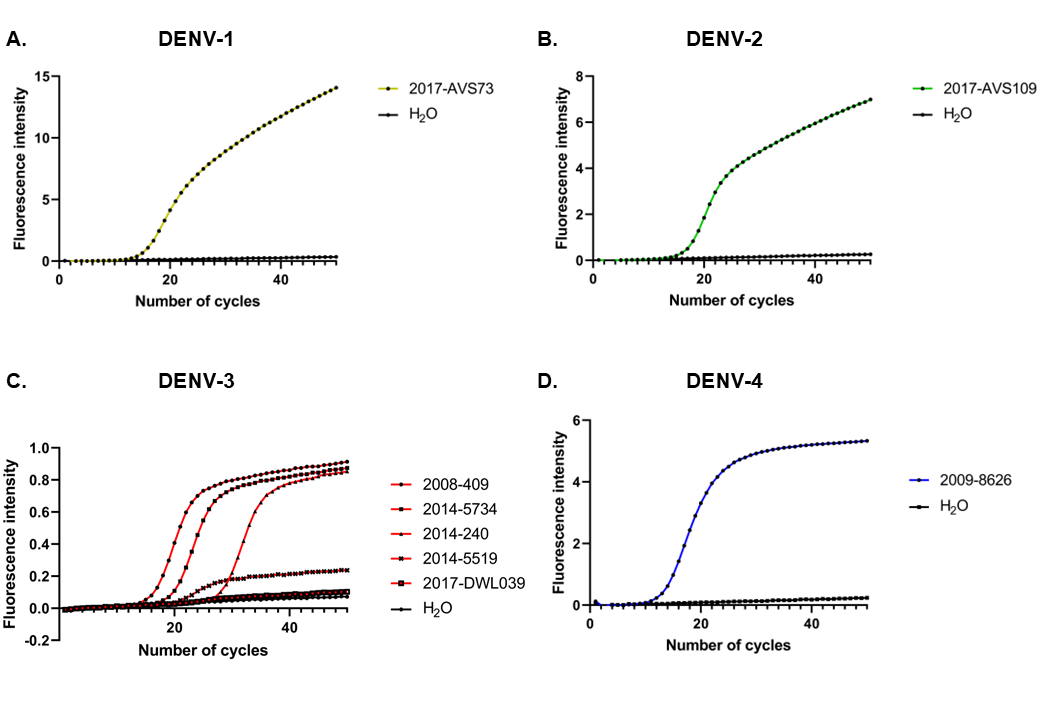

Supplement: S5 Fig — A-D. Fluorescence signal intensity as a function of the number of PCR cycles obtained with the initial primers and probes are shown for DENV-1 (A.), DENV-2 (B.), DENV-3 (C.) and DENV-4 (D.). (TIF) [file pone.0339033.s007.tif]

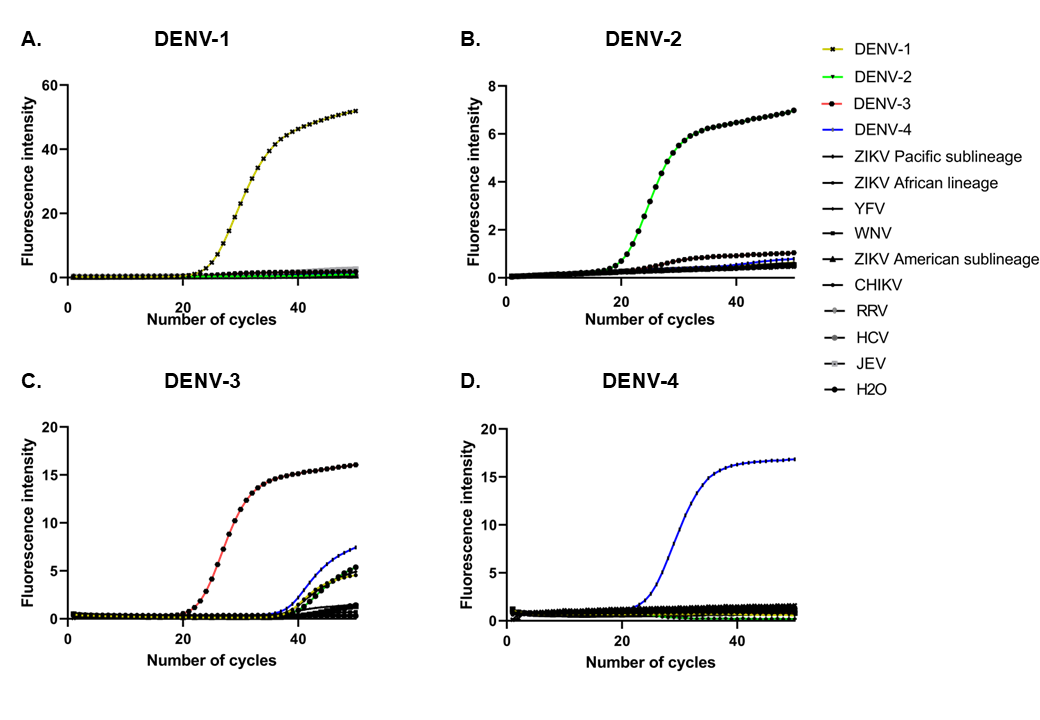

Supplement: S6 Fig — A-D. Fluorescence signal intensity as a function of the number of PCR cycles obtained for DENV-1 to −4, ZIKV, JEV, CHIKV, WNV, YFV, RRV and HCV in the DENV-1 (A.), DENV-2 (B.), DENV-3 (C.) and DENV-4 (D.) channels. (PNG) [file pone.0339033.s008.png]

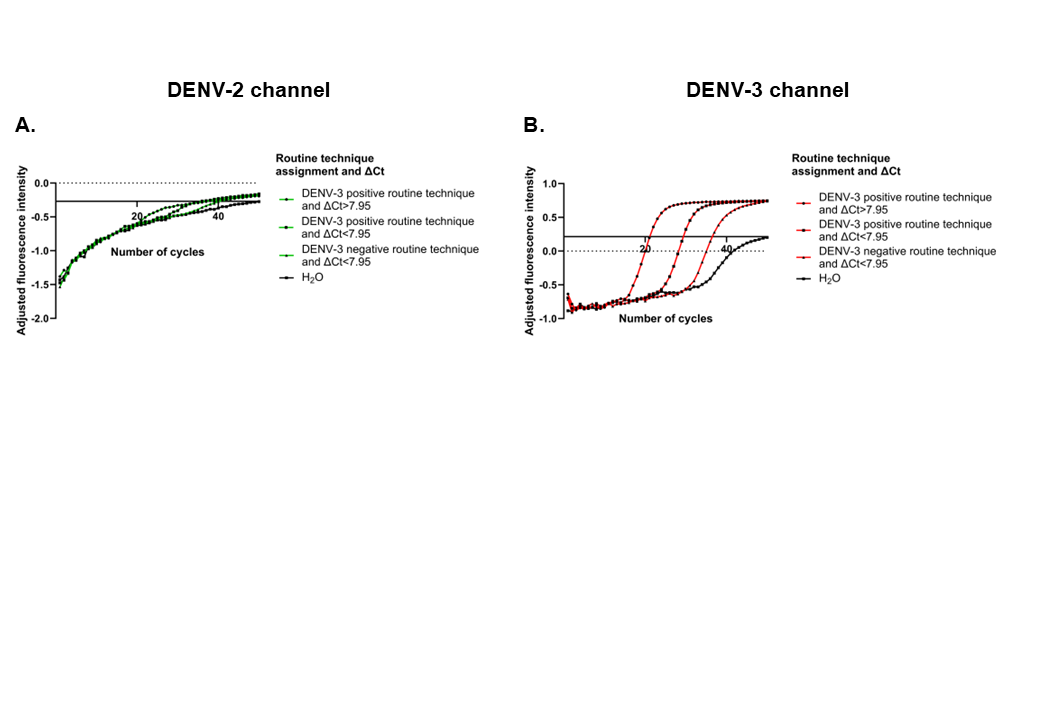

Supplement: S7 Fig — Fluorescence signal intensity as a function of the number of PCR cycles after color compensation and manual threshold adjustment on the negative control H2O are shown for the DENV-2 (A.) and DENV-3 (B.) channels. Intersect of the X axis on the Y axis has been set at the threshold adjusted on the negative control. Round shapes indicate DENV-3 positive samples in the routine technique with a ΔCt > 7.95 between the DENV-2 and the DENV-3 channels in the optimized qRT-PCR, square shapes indicate DENV-3 positive samples in the routine technique with a ΔCt < 7.95 between the DENV-2 and the DENV-3 channels in the optimized qRT-PCR and triangle shapes indicate DENV-3 negative samples in the routine technique with a ΔCt < 7.95 between the DENV-2 and the DENV-3 channels in the optimized qRT-PCR. (TIF) [file pone.0339033.s009.tif]
