# Supplementary material for: Development of a competition assay to assess the in vitro fitness of dengue virus serotypes using an optimized serotype-specific qRT-PCR
Source: PLoS One. 2025 Dec 15;20(12):e0339033. doi: 10.1371/journal.pone.0339033 (PMC12704846; doi:10.1371/journal.pone.0339033)
Supplement: Attachment — Submitted filename: MethodoCompetitionResponseToReviewers20250716.docx [file pone.0339033.s010.docx]

Please find below a point-by-point response to reviewers’ comments.

Additional Editor Comments:

Dear Dr. Catherine Inizan,

Thank you for submitting your manuscript titled "Development of a competition assay to assess the in vitro fitness of dengue virus serotypes using an optimized serotype-specific qRT-PCR" to PLOS ONE.

After careful evaluation by four reviewers, we regret to inform you that your manuscript requires major revisions before it can be considered for publication. Three reviewers have identified significant issues, especially with the methodology and the clarity of the results, which need to be addressed in detail. One reviewer recommended rejection. However, with substantial revisions, we believe the manuscript has the potential to be reconsidered for publication.

We thank the editorial team for this positive appraisal of our work.

Please revise your manuscript to address all the comments and concerns raised by the reviewers. Specifically, focus on the following areas:

Methodology: Provide further clarification and detail regarding the experimental design and analysis. Reviewers highlighted the need for more robust justification for some of the methods used.
Data Interpretation: The presentation and interpretation of your results need to be clearer, particularly in how the data support your conclusions.
Additional Recommendations: Consider the minor recommendations provided by the reviewers to improve the clarity and readability of your manuscript.
Along with your revised manuscript, please include a detailed response letter addressing each of the reviewers' comments, explaining how you have modified the manuscript in response to their suggestions.

If you have any questions or need further assistance, please do not hesitate to contact me.

Best regards,
André Ricardo Ribas Freitas
Academic Editor
PLOS ONE

Reviewer #1: The manuscript by Griffon and colleagues entitled “Development of a competition assay to assess the in vitro fitness of dengue virus serotypes using an optimized serotype-specific qRT-PCR” is interesting research.
In identifying DENV serotype is difficult, when there is a multiple serotype infection. Due to difficulty in identifying; most researchers report single serotype infection rather than multiple serotype infection. This research is quite useful. It will be much better, if the author has validated their claims with the serum sample containing multiple DENV serotypes. Below are the comments that will help to strengthen the manuscript.

Critical comments:
1. In order to avoid discrepancies related to suitability of using serum samples due to viral RNA degradation; it will be best to address the time span in which the samples were collected, stored and reused. Also indicate how these samples were stored in the methods section.

The qRT-PCR was optimized using viruses isolated from serum samples retrieved between 1995 and 2019. In compliance with the reviewer’s recommendation, we validated the qRT-PCR on clinical specimens collected between 1995 and 2019 and stored at -80°C before RNA extraction for the purpose of the current study. We did not observe RNA degradation. We modified the main text as follows:

Line 96-108:

*Human serum samples and viral isolates*

Serum samples were collected for diagnosis purposes between 1995 and 2019 and stored in collection at -80°C at the territorial hospital. Twelve viral isolates obtained from these serum samples were used for the development of the qRT-PCR and the competition assay. Additionally, 8 isolates from arboviruses and an HCV clinical sample were used in a specificity assay and 87 serum samples were used for specificity/sensitivity determination, including five co-infections: DENV-1/DENV-2, DENV-1/DENV-4, DENV-2/DENV-3, DENV-2/DENV-4 and DENV-3/DENV-4. Serum leftovers were retrieved for the current study on July 17, 2020 and April 3^rd^, 2025, (6-30 years after sample collection). RNA was extracted from viral isolates serum samples using the QIAamp Viral RNA extraction kit (Qiagen, Hilden, Germany), following the manufacturer’s instructions. Viral RNAs were stored at -80°C until subsequent use and subjected to detection by qRT-PCR for the current study in 2021-2025.

Line 408-410 “Of note, the Ct did not increase with the duration since sample retrieval, indicating the absence of viral RNA degradation in our collection of serum samples.”

2. The authors have mentioned on the genotypes of DENV-1, 2, 3 & 4 for viral isolates. How the genotypes were identified and by using which gene? Are these from serum samples from your study? Whether these DENV strains were used in Huh7 cultures? Whether these strains were sequenced completely? Include these details in the manuscript.

The serotype of DENV strains used in the current study has been determined either by whole-genome sequencing for strains used for qRT-PCR development and competition assays or by the serotyping qRT-PCR routinely used at the territorial hospital (Johnson et al., J Clin Microbiol, 2005) for clinical specimens. We have given details on the sequencing methods in the Methods section:

Line 121-167

*Serotype and genotype determination*

The serotype and genotype of DENV isolates used in the current study for qRT-PCR development and competition assays in HuH7 cells were been determined by whole-genome sequencing performed on viral RNA extracted from viral cultures, using either an Illumina or an Oxford Nanopore Technologies (ONT) platform.

For sequencing on the Illumina platform, extracted RNA was treated with Turbo DNase (ThermoFisher, Asnières-sur-Seine, France) to digest contaminating cellular DNA. Host rRNA were depleted from RNA samples using the NEBNext® rRNA Depletion kit (New England Biolabs, Évry-Courcouronnes, France) as described previously [23]. RNA from selective depletion was used for cDNA synthesis and Illumina library preparation using the Nextera XT kit (Illumina) with dual indexes and sequenced on an Illumina NextSeq500 (75 cycles, paired-end reads) platform.

Raw paired-end files were processed for removal of Illumina adaptor sequences, trimmed and quality-based filtered using Trimmomatic v0.36 [24]. De novo assembly was performed using metaSPAdes v3.12.0 with default parameters [25]. Scaffolds were queried against the NCBI non-redundant protein database [26] using DIAMOND v 0.9.26 [27]. For each sample, the main scaffold corresponded to DENV and no other viruses were identified. Iterative mapping using CLC-assembly-cell v5.1.0 was used to generate full-length or near full-length consensus genomes followed by manual curation when needed using Geneious Prime v2023. Whole-genome sequences were deposited on the GISAID (<https://gisaid.org/>) EpiArbo database (EPI_SET_240904sd doi: [10.55876/gis8.240904sd](https://urldefense.com/v3/__https:/doi.org/10.55876/gis8.240904sd__;!!JFdNOqOXpB6UZW0!qkGt8W0vn7QHpZK9kby0Vrf_emt9pPjtvI-cb5QOIAsH_MrTuKNtxBBMwD0L53G7wOuCdQ6BfQeggfrtj9CGQum70EFicw$)).

For sequencing on the ONT platform we used the tiling amplicons method for viral RNA which aims to prepare the extracted RNA into different ~1kb amplicons for whole genome sequencing. The first step involves cDNA synthesis through a RT using the Superscript III First Strand Synthesis kit (ThermoFisher Scientific, Carlsbad, CA, USA). Then, we performed a subsequent PCR step using the Q5 Hot Star High-Fidelity DNA polymerase (New England Biolabs, Ipswich, MA, USA) with 15 to 16 primers pairs, selected according to the serotype of the sample and designed in advance to generate amplicons that cover the entire genome. These PCR reactions were split in two pools of amplicons per sample. For each sample, after verifying the amplification with a Tapestation4150, the two pools of amplicons were purified using AMPure XP beads at a ratio of 1.8 and quantified with a Qubit2.0. We then combined the pools and prepared the sequencing library using the ONT Native Barcoding Kit24 V14 (SQK_NBD114.24). Sequencing was performed on a MinION Mk1D with a flow cell R10.4.1 (FLO-MIN114).

Raw POD5 files were processed for base-calling and demultiplexing, and ONT adapter sequence removal using Dorado/0.9.0 (https://github.com/nanoporetech/dorado). Sequencing quality control and analysis of the generated reads were conducted using FastQC/0.11.9 [28] and NanoPlot/1.44.0 (https://github.com/wdecoster/NanoPlot). De novo assembly was carried out using Canu/2.2 (https://github.com/marbl/canu), and the resulting contigs were blasted using BLAST+ (<https://github.com/ncbi/blast_plus_docs>). Reads were aligned to the reference genome with bwa/0.7.17 [29], and near full-length consensus genomes were generated using samtools/1.21 [30] and ivar/1.0.1 (https://github.com/andersen-lab/ivar). The consensus sequences were deposited in GenBank (accession numbers PV791372-PV791376). Table 1 legend indicates the accession numbers of whole-genome sequences generated in the current study. The serotype of clinical specimens used in the specificity/sensitivity assay was determined by the hospital diagnosis laboratory using its routine serotyping qRT-PCR [31].

3. There is no evidence of phylogenetic tree for the mentioned genotypes of DENV-1, 2, 3 & 4. Please provide details in methods and results section.

We built a Maximum Likelihood phylogenetic tree to position the DENV strains used in our study with regards to reported DENV genotypes/serotypes.

We have explained the phylogenetic analyses in the methods section and detailed the results of these phylogenetic analyses in the results section:

Line 168-174:

*Phylogenetic analyses*

A Maximum Likelihood phylogenetic tree showing the genotype/serotype of sequenced strains was built using MAFFT/7.525 [32] for sequence alignment, ensuring high accuracy in the alignment of nucleotides sequences. The resulted aligned sequences were then used as input for the IQ-TREE/2.4.0 [33], which performed model selection and bootstrap analysis to infer the phylogenetic relationship among the strains, providing a robust framework for understanding their evolutionary history. The phylogenetic tree was visually edited using iTOL v7.2 [34].

Line 306-309

The serotype and genotype of DENV strains used for qRT-PCR development have been determined by whole-genome sequencing. Positioning of these strains in the context of the global DENV diversity are shown in a phylogenetic tree (Supplementary figure 1). Serotypes are summarized in Table 1.

4. How many serum samples were used in this experiment? How long the serum samples were stored before RNA extraction? How these samples were stored? Indicate in which experiments these samples has been used? Include this information at appropriate places in methods section.

We used twelve viral isolates for technical development of the qRT-PCR and the competition assay and 8 viral isolates, an HCV clinical sample and 87 DENV clinical samples for specificity validation of the qRT-PCR. We have specified in the methods section how these samples have been used and how long serum samples were stored before RNA extraction. Table 1 summarizes the applications in which assay each of the twelve viral isolates have been used.

Line 96-108:

*Human serum samples and viral isolates*

Serum samples were collected for diagnosis purposes between 1995 and 2019 and stored in collection at -80°C at the territorial hospital. Twelve viral isolates obtained from these serum samples were used for the development of the qRT-PCR and the competition assay. Additionally, 8 isolates from arboviruses and an HCV clinical sample were used in a specificity assay and 87 serum samples were used for specificity/sensitivity determination, including five co-infections: DENV-1/DENV-2, DENV-1/DENV-4, DENV-2/DENV-3, DENV-2/DENV-4 and DENV-3/DENV-4. Serum leftovers were retrieved for the current study on July 17, 2020 and April 3^rd^, 2025, (6-30 years after sample collection). RNA was extracted from viral isolates serum samples using the QIAamp Viral RNA extraction kit (Qiagen, Hilden, Germany), following the manufacturer’s instructions. Viral RNAs were stored at -80°C until subsequent use and subjected to detection by qRT-PCR for the current study in 2021-2025.

5. How the primers/probes were designed? Which gene was used for designing the primers/probes? Whether the designed primers/probes were from gene/genome sequences available from data repository or from your own sequencing or from other researcher’s publication? Please, include the details appropriately.

Primers/probes developed for the current study were adapted from a published qRT-PCR from Ito et al. We specified it in the results section:

Line 309-313: We optimized an existing singleplex serotype-specific qRT-PCR [22] used for DENV serotyping in diagnostic laboratories. Primers and probes from this qRT-PCR were coupled with a combination of fluorochromes compatible with multiplexing on the LightCycler 480 (Table 2): DENV-1 probe was coupled to FAM, DENV-2 probe to Yakima Yellow, DENV-3 probe to ROX and DENV-4 probe to Cy5 (Table 2).

Primers and probes from all four DENV serotypes align to the E gene. We specified in the results section how we performed the alignments:

Line 313-316: “Alignment of these primers and probes with a set of published sequences (detailed in Supplementary table 1) showed that primers and probes for the four DENV serotypes are located in the E gene (Figure 1A).

And Line 324-335:

We aligned DENV-3 primers and probes with DENV-3 sequences from genotype I, II and III including published sequences and sequences of DENV-3 genotype I strains retrieved in 2017 in New Caledonia generated in the framework of the current study. This alignment evidenced a perfect match for the forward primer (Figure 1B). However, this alignment revealed two mismatches with DENV-3 strains retrieved in New Caledonia in 2017 and in Malaysia: a mismatch at position 8 in the probe (C in DENV-3 VS T in the probe, Figure 1C) and a mismatch at position 17 in the reverse primer (A in the DENV-3 VS G in the reverse complemented sequence of the reverse primer, Figure 1D). Sequences of the probe and reverse primer were optimized according to these observations to include degenerated nucleotides able to detect the diversity of DENV-3 strains at these positions: a Y was introduced in the probe sequence at position -8, and a Y was introduced in the reverse primer sequence at position 17.

6. Competition assay – As per the methods, 2 x 105 HuH7 cells were seeded and after 24 hours viral isolates were introduced. Whether these cells attain confluence in 24 hrs? The cell population might also affect the titer value of viral load, right? How this was avoided?

A seeding concentration 2x10^5^ cells/well was determined in order to reach confluence at 24h. We considered a doubling time of 24h for HuH7 cells and assumed to reach 4x10^5^ cells per well 24h after seeding. This number of cells was used to calculate the multiplicity of infection of 1. We specified in the Methods section:

Line 278-279: “HuH7 were seeded at 2 x 10^5^ cells per well in 12-well plates. We assumed a doubling time of 24h for HuH7 cells [36], targeting 4 x 10^5^ cells to be infected in each well 24h after seeding.”

Line 283-285: “Multiplicities of infection (MOI) were calculated for the 4 x 10^5^ cells/well based on the titer of the viral isolate in FFU/mL determined by Immunofluorescent Focus Assay.”

7. The result section has no way to identify whether experimental observation is from human serum or cell culture. Please indicate wherever appropriate.

We specified the origin of the samples, either from serum sample or viral isolate, in any needed occurrence as follows:

Line 317-318 “RNA extracted from viruses isolated in culture from serum samples collected in”

Line 320-321 “DENV-3 RNA extracted from viruses isolated in culture from serum samples collected in”

Line 338-339 “DENV-1 to DENV-4 RNA extracted from viruses isolated in culture from serum samples collected”

Line 345-346 “on DENV RNAs extracted from viruses isolated in culture from serum samples.”

Line 363 “isolates”

Line 416 “isolates”

Line 416-417 “retrieved from infected C6/36 cultures”

Line 429 “single viral isolate produced by infected C6/36 cells.”

Line 436-437 “DENV viral isolates from C6/36 cells,”

Line 454 “DENV viral isolates from C6/36 cells”

8. The competition assay shown interesting results. Whether the author validated in human serum samples? It is requested the author should validate the competition assay observation in their collected human serum samples containing one or more DENV serotypes.

The competition assay was not intended to identify the infecting serotype in a primary human serum sample. It was rather designed to compare the replicative fitness of two DENV strains isolated in cell culture. Both strains have to be introduced in known concentrations. It is therefore required to titrate these strains beforehand, in order to determine their concentrations in infective viral particles. These concentrations in FFU/mL are determined by titration on C6/36 cells revealed by immunofluorescence. Titration of co-infected human serum samples requires the use of serotype-specific primary antibodies to distinguish between the two DENV strains present in the human serum samples and determine each titer. The affinity, avidity and specificity of serotype-specific anti-DENV antibodies are variable from one serotype to the other and would therefore not allow to precisely determine the infectious titer of two DENV strains in a co-infected human serum sample. Furthermore, our assays rely on a MOI of one for each DENV strain introduced in the competition assay, which is difficult to attain with primary human serum samples, which are less concentrated than viral isolates.

For all these reasons, our competition assay is not suitable for the comparison of the replicative fitness of two DENV strains contained in a human primary serum sample.

9. Whether competition assay can help in identifying different genotypes within serotypes? Explain in the discussion section.

The competition assay was not intended to identify the infecting serotype/genotype in a primary human serum sample. This assay rather aims at assessing and comparing the replicative fitness of two DENV viral isolates belonging to different serotypes. The most efficient approach to identify the infecting genotype within serotypes remains sequencing. However, development of a genotype-specific qRT-PCR, as in O’Connor et al, EMI, 2021 could allow to compare the replicative fitness of two viral isolates belonging to different genotypes within the same serotype.

10. The discussion is superficial. It will be better to do an in-depth discussion. Compare your observation with other researchers’ article and discuss.

We have enriched the discussion with a paragraph presenting DENV serotyping by qRT-PCR (line 476-483), a paragraph highlighting the importance of DENV serotyping (line 491-511), a paragraph referencing studies of DENV fitness (line 512-520) and a paragraph reporting competition assays for DENV and other viruses (line 521-542), deepening the discussion of our results in perspective with available literature.

11. The authors have provided two genome data from GenBank. What is the purpose of providing it? If you used in your experiments, provide where and how. Similarly, you have provided data from GISAID, please include in which experiment you have used it or what is the importance of these sequence? Include at appropriate places.

Strains used in the current study were whole-genome sequenced to confirm serotype/genotype/lineage, which meets reviewer’s comment 2. This sequencing allowed to identify the serotype and genotype of DENV strains used in the current study. Confirming serotype of strains used in qRT-PCR development is crucial to validate the assay. These sequences were deposited on GenBank or GISAID. These deposited sequences were used to build the phylogenetic tree shown in Supplementary figure 1. We have specified in the methods section:

Line 121-125:

*“Serotype and genotype determination*

The serotype and genotype of DENV isolates used in the current study for qRT-PCR development and competition assays in HuH7 cells were been determined by whole-genome sequencing performed on viral RNA extracted from viral cultures, using either an Illumina or an Oxford Nanopore Technologies (ONT) platform.”

Line 139-141:

Whole-genome sequences were deposited on the GISAID (<https://gisaid.org/>) EpiArbo database (EPI_SET_240904sd doi: [10.55876/gis8.240904sd](https://urldefense.com/v3/__https:/doi.org/10.55876/gis8.240904sd__;!!JFdNOqOXpB6UZW0!qkGt8W0vn7QHpZK9kby0Vrf_emt9pPjtvI-cb5QOIAsH_MrTuKNtxBBMwD0L53G7wOuCdQ6BfQeggfrtj9CGQum70EFicw$)).

Line 162-165:

“The consensus sequences were deposited in GenBank (accession numbers PV791372-PV791376). Table 1 legend indicates the accession numbers of whole-genome sequences generated in the current study.”

Use of deposited sequence in phylogenetic analyses is specified Line 168-170:

“*Phylogenetic analyses*

A Maximum Likelihood phylogenetic tree showing the genotype/serotype of sequenced strains was built”

12. Since the serum samples from before and after COVID pandemic i.e., from 1995-2021 was included in your study, did the author perform any COVID testing in these samples? If so, is there any difference in DENV viral load or serotype difference in COVID positive serum samples? It is advised to include COVID analysis status (as acknowledgement or wherever possible) in this manuscript which might be helpful for the researchers to know whether COVID has impact on DENV infection.

We apologize for the inaccuracy of the sample retrieval dates. The study was conducted on strains collected in 1995-2019, during the pre-COVID-era. Dates have been modified in all occurrences.

Specific comments:
Line 104-106: how the genotypes were identified? Provide details.

As mentioned above, we added details on the genotype identification in the Methods section:

Line 121-125

*“Serotype and genotype determination*

The serotype and genotype of DENV isolates used in the current study for qRT-PCR development and competition assays in HuH7 cells were been determined by whole-genome sequencing performed on viral RNA extracted from viral cultures, using either an Illumina or an Oxford Nanopore Technologies (ONT) platform.”

And Line 164-167:

“Table 1 legend indicates the accession numbers of whole-genome sequences generated in the current study. The serotype of clinical specimens used in the specificity/sensitivity assay was determined by the hospital diagnosis laboratory using its routine serotyping qRT-PCR [31].”

Line 114: Provide the primer/probe list as supplementary data.

Sequences of the primers and probes used in the current study are given in Table 2. This is specified:

Line 196-197

Primers and probes sequences are given in Table 2.

And line 218-219:

Initial [22] and optimized primers and probes (Eurogentec, [Seraing, Belgique](https://www.google.com/search?sca_esv=80a4d258620f7496&sca_upv=1&rlz=1C1CHBF_frNC955NC955&sxsrf=ADLYWIJMAcccjGnfHKornEwREuQ48PETGA:1719446051056&q=Seraing&si=ACC90nzx_D3_zUKRnpAjmO0UBLNxnt7EyN4YYdru6U3bxLI-L-3XK9kA-xn6rmcH02IsEnBNtKXnsHW380ctfkaxHvcrbEQQTxNj0u3qdsG6FMv7dA-R0-ipSmD4dqeNSGqylUrlsK_s-X4E6i6-AZn8mX9cpF6xsvGwQIlHwUhSvgEVqdhxW-10vLZWvqtZ1nryhlydSfJ1&sa=X&sqi=2&ved=2ahUKEwjSpNLYu_qGAxXIU2wGHSiFBdwQmxMoAHoECDAQAg)) are detailed in Table 2.

Line 240-242: The sentence ended with a citation. From the information, it is a statement from your observation. Avoid providing citation for your statements unless you have already published article replated to this. It is suggested that the author should reframe the sentence.

The absolute concentration of RNA from DENV-1 to -4 have been determined using the qRT-PCR described in the cited reference. We have specified it in the sentence which is now line 362-364:

“The absolute concentration in genome copies per µL of four RNA extracts from the DENV-1 to -4 isolates were determined by absolute quantification using the qRT-PCR described in [21].”

Line 114 & 203: How many sets of primers/probes were used? The authors have mentioned two different articles (Warrilow et al., 2002, Ito et al., 2004) as reference. Clarify and rewrite.

The qRT-PCR published by Warrilow et al. was used in our study to determine the absolute concentration in RNA copies number/µL of the DENV strains contained in the RNA extract from serum samples used in the current study. The qRT-PCR published by Ito et al. is serotype-specific. It was however initially singleplex and unable to detect some DENV-3 strains. We optimized it in the current study to multiplex it and optimize DENV-3 detection. We have specified:

Line192-193 This qRT-PCR is pan-serotypic and allows the absolute quantification of DENV RNA copies number per µL.

Line 216-218 This qRT-PCR was initially singleplex and serotype-specific but did not allow for the absolute quantification of DENV RNA copies number per µL for each serotype.

Line 309-310 We optimized an existing singleplex serotype-specific qRT-PCR [22] used for DENV serotyping in surveillance.

Table 2: Lacks information on gene, gene location, repository name and ID from which the sequence was obtained.

Positions of the E gene amplified and detected by the different combinations of primers and probes were specified in Figure 1. We have added them to Table 2. Sequences used for DENV-3 primers and probe optimization are given in Figure 1. We have specified this in Figure 1 Legend. Their GenBank accession numbers are now specified in Supplementary table 1.

The preprinted supporting methods section does not provide any valid information, it is just the repetition of the manuscript. Avoid submitting this or provide valid information for supporting additional methods which are not provided in the main manuscript.

We apologize for the redundancy. Submitting the preprint when existing is a request from PLOS One.

Reviewer #2: This is an interesting manuscript but the authors need to make clear for the reviewers and all readers.
1. The authors described that the authors developed a new multiplex RT-PCR system for quantification of DENV for competition assay. Did the authors make validation about the cross reactivity f the primers and probes with other serotypes of the virus as well as the other flaviviruses. Please describe clearly at the revised manuscript.

We thank the reviewer for this suggestion. We have performed a specificity validation of our assay using viral RNAs from other arboviruses using the three lineages from ZIKV, as well as viral RNA from CHIKV, RRV, YFV, WNV, JEV and HCV. The qRT-PCR we described was highly specific and showed no cross-detection of other arboviruses in either of the 4 channels dedicated to DENV serotypes detection.

We have specified in the Methods section Line 259-270:

*“Specificity validation*

Viral isolates of Zika virus (ZIKV) DAK 84 (African lineage), ZIKV MRS_OPY_Martinique (Asian lineage, American sub-lineage), ZIKV 5132 (Asian lineage, Pacific sub-lineage), Chikungunya virus (CHIKV) NC/2011-568 (East-Central-South-African lineage), Ross River Virus from the French National Reference Center for arboviruses (RRV), West-Nile Virus from the French National Reference Center for arboviruses (WNV), the vaccine strain (Stamaril®) from Yellow Fever Virus (YFV), the vaccine strain (CD Jevax®) for Japanese Encephalitis (JEV) along with a serum sample positive for hepatitis C virus (HCV) were used in the specificity assay. Viral RNA was extracted from these samples using the QIAamp Viral RNA extraction kit (Qiagen, Hilden, Germany), following the manufacturer’s instructions. Viral RNA were stored at -80°C until subsequent use in the optimized serotype-specific qRT-PCR developed in the current study.”

We have added to the Results section Line 379-387:

“*Evaluation of the specificity and sensitivity of the multiplex serotype-specific qRT-PCR*

To assess the specificity, the fourplex serotype-specific qRT-PCR was tested against a panel of viruses including DENV-1 to -4, ZIKV, JEV, CHIKV, [WNV](https://www.sciencedirect.com/topics/biochemistry-genetics-and-molecular-biology/west-nile-virus), [YFV](https://www.sciencedirect.com/topics/pharmacology-toxicology-and-pharmaceutical-science/yellow-fever-virus), RRV and [HCV](https://www.sciencedirect.com/topics/agricultural-and-biological-sciences/hepatitis-c-virus). The DENV-1 to -4 RNAs were correctly detected by Atto425, Atto590, FAM and Cy5 signals (Supplementary figure 6). The qRT-PCR did not generate any signal for ZIKV, JEV, CHIKV, [WNV](https://www.sciencedirect.com/topics/biochemistry-genetics-and-molecular-biology/west-nile-virus), [YFV](https://www.sciencedirect.com/topics/pharmacology-toxicology-and-pharmaceutical-science/yellow-fever-virus), RRV, and HCV, except for DENV-1, -2, -4 and YFV which generated residual signal with Ct≥ 40 in the FAM channel, far above the detection limit of the fourplex qRT-PCR for DENV-3 which is ~34 cycles as determined in the sensitivity assay (Supplementary figure 4). The negative control without RNA [template](https://www.sciencedirect.com/topics/biochemistry-genetics-and-molecular-biology/dna-template) was also negative in the four channels.”

2. Regarding the ethical issues, did the authors take written informed consent from the patients or please briefly describe about the justification for not taking ethical clearance.

This research relied on the secondary use of serum samples initially collected for diagnosis purposes. This research complies with the French regulation for Research Involving the Human Person of Category 3 (RIPH3) and as such does not require written informed consent. Participants were informed of this possible secondary use for research purposes and were given the opportunity to object to this use.

Ethics statement was moved to the beginning of the Methods section Line 88-95

*“**Ethics statement*

The current study was conducted in compliance with the Declaration of Helsinki principles. Serum samples collected in 1995-2019 came from laboratory-confirmed dengue patients who were informed of and did not object to the secondary use of their serum sample for research purposes. This study reusing serum samples received administrative and ethical clearance in France from the “Comité de Protection des Personnes Sud-Est II” (n° ID-RCB 2019-A03114-53, n° CPP 19.12.06.49357) and from the Consultative Ethics Committee of New Caledonia. The study was recorded on Clinicaltrials.gov (ID: NCT04615364).”

3.The authors described the the concentration of the virus by mixing supernatants and the authors described that the results showed expected results. The authors should make confirmation of the results with another method such as immunofluorescence based focus assay method and the authors already established this system.

DENV produces large amounts of defective particles, which are variable across different strains. Genomes of these defective particles can be detected by qRT-PCR but do not correlate with infectious titers, as measured by immunofluorescence for instance. This can be observed in Figure 4C: all DENV strains have been introduced at a MOI of 1 in the inoculum (as determined by immunofluorescence). However, their concentrations in genome copies are far from being 1:1. For instance, we detected 85.2% of DENV-2 in the inoculum of the DENV-1:DENV-2 mix.

Furthermore, quantifying two serotypes in a mixture would require serotype-specific antibodies with equal affinity, which are currently not commercially available.

For these reasons, we feel that immunofluorescence is not an indicated method to confirm the capacity of the optimized serotype-specific qRT-PCR to accurately quantify viral concentrations in mixture of strains from two serotypes.

We confirmed the accuracy of our optimized serotype-specific qRT-PCR by measuring the concentrations in genome copies of each DENV strain introduced in mixes at ratios 0:100, 25:75, 50:50, 75:25 and 100:0 and showed that detected concentrations are consistent with the concentrations we targeted when mixing the two viral supernatants.

4. Regarding the competition assay, the authors must clear describe how many and how did the author check the competition assay such DENV1 and DENV-2 or DENV1 and DENV-3 or DENV2 and DENV-4. This point did not clear to the reviewers.

We apologize for this lack of clarity. We have specified in the text

Line 437-444:

“More specifically, the DENV-1 viral isolate AVS94 was mixed with either the DENV-2 viral isolate DWL-089 or the DENV-3 viral isolate DWL-039. The DENV-2 viral isolate DWL-089 was also mixed with the DENV-3 viral isolate DWL-039 (Table 1). Competition assays were performed in three independent replicates. Ratio calculation was based on the titer of viral suspension determined by immunofluorescent focus assay. Concentrations of each viral isolate in the inoculum and the viral progeny at three days post-infection (culture supernatant) were determined by applying the quantitative multiplex serotype-specific RT-PCR developed here.”

5. The authors did not describe how many samples were used in this study .

This comment is similar to Reviewer 1 comment 2. Manuscript now reads:

Line 96-103:

“*Human serum samples and viral isolates*

Serum samples were collected for diagnosis purposes between 1995 and 2019 and stored in collection at -80°C at the territorial hospital. Twelve viral isolates obtained from these serum samples were used for the development of the qRT-PCR and the competition assay. Additionally, 8 isolates from arboviruses and an HCV clinical sample were used in a specificity assay and 87 serum samples were used for specificity/sensitivity determination, including five co-infections: DENV-1/DENV-2, DENV-1/DENV-4, DENV-2/DENV-3, DENV-2/DENV-4 and DENV-3/DENV-4.”

6. Why did the author used HuH cells for competitive assess and please briefly describe the justification.

In its human host, DENV mostly replicates in cells from the monocytic lineage and hepatocytes. The human hepatocellular carcinoma cell line HuH7 thus represents a physiological cellular model for DENV replication in its human host. We have specified line 429-431:

“As in humans, DENV mostly replicates in hepatocytes and cells from the monocytic lineage [38], the hepatocellular carcinoma cell line HuH7 was used as a physiological cellular model for DENV replication in its human host.”

7. For the competition assay, did the authors check different genotypes within one DENV serotypes. How did you do for two different genotypes competition assay because your RT-PCR system did not support for this competition assays,

The current competition assay has been developed to compare the replicative fitness of two DENV strains belonging to two different serotypes. The multiplex serotype-specific qRT-PCR developed here allows to discriminate between and quantify two strains from two serotypes and not between two genotypes within one serotype. Other qRT-PCR have been developed to quantify genotypes within one serotype (O’Connor et al, EMI, 2021). We have added this limitation to the discussion section:

Line 570-573: “Fifth, the current assay combined with the serotype-specific qRT-PCR we optimized does not allow to compare the replicative fitness of two strains belonging to two different genotypes within the same serotype. However, it can be easily adapted to compare the fitness within genotypes by applying genotype-specific qRT-PCR, as developed in [48]. “

Reviewer #3: This manuscript tried to introduce a novel competition assay of dengue virus serotypes using an optimized serotype-specific qRT-PCR. My major concerns are as follows:
1. The authors should provide more detailed results to support the specificity of the assays. From Figure 3, I could infer that the manual threshold adjustment is not reliable. Because fluorescence intensity could be influenced by many factors not restricted to the matching degree between the primers and the template. However, the delta Cp value between matched and unmatched primer pairs is relatively reliable. Recommend the authors set up a reliable threshold of the delta Cp value to support the specificity.

We thank the reviewer for this suggestion. We observed that, when the threshold is adjusted on the negative control, the average delta Ct value of the DENV-3 positive control detected in the DENV-2 Atto590 channel and the DENV-3 FAM across 25 independent experiments is 7.95 ± 3.88. Therefore, an alternative approach would be to consider any signal above 7.95 added to the Ct of DENV-3 positive control detected in the DENV-2 channel as positive. Analysis of our data with this rule confirms the correct serotype assignment of 11/11 samples determined as DENV-3 positive by the hospital routine serotyping technique. In addition, analysis of our data with this rule allows to identify DENV-3 as the infecting serotype in 2 samples identified as negative by the routine technique. Finally, careful observation of the amplification curves as shown in Supplementary Figure 7 allows to identify DENV-3 as the infecting serotype in 2 samples identified as DENV-3 positive by the routine technique and 6 samples identified as negative by the routine technique. Only one sample typed as DENV-2 by the routine technique was identified as DENV-3 positive by our technique, yielding one false positive. The serotype-specific qRT-PCR proposed here thus seems more sensitive at serotyping equivocal samples.

We have added Line 352-355:

When the threshold was adjusted on the negative control, the average ΔCt value between the Ct of DENV-3 positive controls detected in the DENV-2 Atto590 channel and their Ct detected in the DENV-3 FAM channel across 25 independent experiments was 7.95 ± 3.88.

Line 401-411:

Analysis of this specificity/sensitivity assay with threshold adjustment on the negative control and considering DENV-3 positive any sample with a Ct in the DENV-3 channel above 7.95 added to the Ct detected in DENV-2 channel correctly assigned 11/11 samples as compared to the routine technique. In addition, DENV-3 was identified as the infecting serotype in two samples identified as negative by the routine technique. Finally, analysis of the amplification curves (Supplementary figure 7) allowed to identify DENV-3 as the infecting serotype in two samples identified as DENV-3 positive by the routine technique and 6 samples identified as negative by the routine technique. Only one sample typed as DENV-2 by the routine technique was identified as DENV-3 positive by our technique, yielding one false positive.

2. Could the author explain why the efficiencies of the qRT-PCR are so high and looks unnormal?

Efficiency figures we gave actually correspond to amplification factors. We apologize for the confusion. They were derived from the standard curves and automatically calculated by the LightCycler480 software. Manual re-calculation of amplification factors gives similar results.

We calculated efficiencies as “Efficiency” = -1 + “amplification factor” and added them in the text line 375-377:

“qRT-PCR amplification factor was 1.993, 1.975, 1.902, 1.898 for DENV-1, -2, -3 and -4 respectively. qRT-PCR efficiency was 99.3%, 97.5%, 90.2% and 89.8%. Linearity (R²) was 0.99975, 0.99975, 0.9985, 0.99965 for DENV-1, -2, -3 and -4 respectively.”

3. The authors should enroll more samples to validate the performance of the developed assays. and provide the related statistical analysis.

We thank the reviewer for this suggestion. We have included 87 clinical specimens in a specificity/sensitivity assay, including 32 DENV-1, 20 DENV-2, 13 DENV-3, 7 DENV-4, a DENV-1/DENV-2, a DENV-1/DENV-4, a DENV-2/DENV-3, a DENV-2/DENV-4 and a DENV-3/DENV-4 co-infection and 10 negative samples according to the reference technique.

The optimized qRT-PCR proposed here correctly assigned 30 DENV-1, 19 DENV-2, 13 DENV-3 and 7 DENV-4 for a total of 69 correct assignments, no false positives, 9 samples identified as DENV-3 positive while being typed as negative by the routine technique and three false negatives. Regarding the co-infections, while the routine technique detected two co-infections (DENV-2/DENV-3 and DENV-2/DENV-4) out the five co-infections historically determined at the time of sample collection; the current optimized qRT-PCR detected three co-infections (DENV-1/DENV-4, DENV-2/DENV-3, DENV-3/DENV-4).

We have added to the Methods section:

Line 100-103:

“Additionally, 8isolates from arboviruses and an HCV clinical sample were used in a specificity assay and 87 serum samples were used for specificity/sensitivity determination, including five co-infections: DENV-1/DENV-2, DENV-1/DENV-4, DENV-2/DENV-3, DENV-2/DENV-4 and DENV-3/DENV-4.”

Line 165-167:

“The serotype of clinical specimens used in the specificity/sensitivity assay was determined by the hospital diagnosis laboratory using its routine serotyping qRT-PCR [31].”

And the Results section line 388-400:

Eighty-seven serum samples from DENV patients were included in a specificity/sensitivity assay, including 32 DENV-1, 20DENV-2, 13 DENV-3, 7DENV-4, one DENV-1/DENV-2, DENV-1/DENV-4, one DENV-2/DENV-4, one DENV-2/DENV-3 and DENV-3/DENV-4 and 10 negative samples according to the routine technique [31]. The optimized qRT-PCR correctly assigned 30 DENV-1, 19 DENV-2, 13 DENV-3 and 7 DENV-4 for a total of 69 correct assignments, no false positives, 9 samples identified as DENV-3 positive while being typed as negative by the routine technique and three false negatives, yielding 93.75% sensitivity, 100% specificity for DENV-1, 95% sensitivity, 100% specificity for DENV-2, 100% sensitivity, 86.95% specificity for DENV-3 and 100% sensitivity, 100% specificity for DENV-4. Regarding the co-infections, while the routine technique detected two co-infections (DENV-2/DENV-3 and DENV-2/DENV-4) out the five co-infections historically determined at the time of sample collection, the current optimized qRT-PCR detected three co-infections (DENV-1/DENV-4, DENV-2/DENV-3, DENV-3/DENV-4).

Reviewer #4: Introduction
- Line 44 - 45: DENV serotypes share between 34 and 73% identity at the nucleotide sequence level [Blok J et al., 1985].
Please check ([https://doi.org/10.1186/s12864-019-6311-z](https://urldefense.com/v3/__https:/doi.org/10.1186/s12864-019-6311-z__;!!JFdNOqOXpB6UZW0!p-JK5mybjXP_f1Ti0Q_4ScaQRbLm_qE4_Hoje1QPG0DsiVcHhssbfL3ajJgM2fASvGH5fBClBuzGjhO3EA$)). Inter-serotype nucleotide identity is around ~60-70% while polypeptide sequence identity might be as low as ~30%.
Thank you for pointing out this inaccuracy. We changed the text and the reference associated:

Line 45

“DENV serotypes share between ~60-70% identity at the nucleotide sequence level [1, 2]”

-Line 47-48: However, the mechanisms underlying DENV serotypes co-circulation and replacements are misunderstood.
To be misunderstood could mean "to fail to understand somebody/something correctly." Please consider using another word or rephrase the sentence.
We changed misunderstood line 50 by “remain elusive”.

-Line 48-51: Viral replicative fitness may contribute to DENV serotype replacements. It is therefore of prime importance to set up experimental models to assess the relative replicative fitness of strainsfrom different serotypes.
DENV serotype replacements could not be primarily attributed to replicative fitness. Host immune response and herd immunity might play more important roles in serotype replacements [[https://doi.org/10.1073/pnas.1120621109](https://urldefense.com/v3/__https:/doi.org/10.1073/pnas.1120621109__;!!JFdNOqOXpB6UZW0!p-JK5mybjXP_f1Ti0Q_4ScaQRbLm_qE4_Hoje1QPG0DsiVcHhssbfL3ajJgM2fASvGH5fBClBuyarfxcGg$)]. Please provide more references in the introduction or the discussion to convince that replicative fitness evidently contribute to serotype replacements in human (or mosquito) population. If there is no evidence, please at least provide a use case for assaying replicative fitness in vitro that could contribute to the field (e.g. vaccine or drug development).
Herd immunity is indeed an important contributor to serotype replacements. Some case studies however suggest that other drivers may contribute to DENV serotype replacements.

For instance, following a co-circulation event in New Caledonia, we observed the concomitant expansion of DENV-2 and extinction of DENV-3, which last caused outbreaks in 1997 and 1995 respectively. An entire generation of New Caledonia population was thus fully naïve for both serotypes when they were reintroduced thirty years later. In 2017, New Caledonia experienced an important DENV outbreak: DENV-1 was the major circulating serotype, and co-circulated with DENV-2 and -3. In 2018, while DENV-2 became dominant, DENV-1 circulation decreased, DENV-3 circulation came to an end. DENV-2 then circulated in New Caledonia until 2021. As population immunity was similar to both serotypes when introduced, this observation suggests that factors other than herd immunity contribute to DENV serotype replacements.

Furthermore, enhanced replicative fitness in the mosquito vector was observed in New Caledonia upon a genotype replacement of DENV-1 strains (O’Connor et al., EMI, 2021).

In addition, a larger DENV-2 outbreak in Taiwan was associated with viral evolution since the previous outbreak. Acquisition of the NS5V357E mutation resulted in enhanced replicative fitness in vitro in mosquito and mammalian cells (Ko et al, iScience, 2024).

Contribution of viral fitness to serotype replacements is thus worth assessing.

We discussed this importance further:

Line 512-520:

To improve dengue outbreak prevention and the surveillance of antiviral resistance [46], it is crucial to strengthen genomic surveillance but also to better assess viral replicative fitness [47]. For instance, a larger DENV-2 outbreak in Taiwan was associated with viral evolution since the previous outbreak. Acquisition of the NS5V357E mutation resulted in enhanced replicative fitness *in vitro* in mosquito and mammalian cells [6], highlighting the importance to assess viral replicative fitness. Similarly, emergence of Asian I genotype of DENV-2 in Viet-Nam was associated with enhanced in vivo fitness denoted by higher viraemia in patients [48]. Furthermore, enhanced replicative fitness in the mosquito vector was observed in New Caledonia upon a genotype replacement of DENV-1 strains [41].

And Line 523-530:

*In vitro* competition fitness assays are essential tools for evaluating viral replication capacity, offering valuable insights into viral evolution, such as the role of HIV-specific mutations [58], while deepening our understanding of pathogenic mechanisms for adenoviruses for example [58], to provide insights into replicative fitness and transmission between different hosts, for instance among emerging WNV strains [59] and to unravel the mechanisms of competition and replacement of epidemic SARS-CoV-2 variants [60], or to contribute to the optimization of treatment strategies [61], for instance for the monitoring of drug-resistant influenza strains [62, 63].

-Line 52-53: The study of viral replicative fitness has become an expanding field of research [Wargo et al., 2012].
The referenced paper was from 2012 (>10 years ago). Please provided a newer reference that the study of viral replicative fitness is still relevant today, especially in dengue research.

Line 55 We have added a recent citation (Nov 2024) which studied the replicative fitness of an emergent DENV strain with regard to its epidemiologic and pathogenic potential.

<https://www.sciencedirect.com/science/article/pii/S2589004224021242>

Materials and Method

-Please add a "statistical analyses" part/sub-section that at least includes

We have added a “Statistical analyses” section to the Material and methods line 295-303.

1) Sample size calculation (How do you justify that the number of samples or repeats are enough to accurately evaluate your assay?

As per reviewer’s 1 recommendation, we have performed complementary sensitivity/specificity assessment by subjecting 96 RNA samples from other arboviruses and clinical specimens to the multiplex serotype-specific qRT-PCR we developed in the current study. A sample size of at least 95 gives a 99% confidence to detect a 99% sensitivity/specificity +/- 2% (doi: [10.1016/B978-0-12-814515-9.00053-9](https://doi.org/10.1016/B978-0-12-814515-9.00053-9)). We have specified it Line 296-298 “Ninety-six RNA extracts, including 8 RNA extracts from arbovirus isolates, an HCV clinical sample and 87 serum samples from patients were analyzed by qRT-PCR in the sensitivity/specificity assay. A sample size above 95 gives a 99% confidence to detect a 99% sensitivity/specificity ± 2%[37].”

2) Describe statistical tests used in this study. If possible, explain why the tests were selected. For example, chi-square tests were used for testing the difference in proportions. Why Chi-square tests was preferred to Fisher's test

We have specified Line 299-302 “As a non-parametric test typically used for testing independence for 2×2 contingency tables, Fisher’s exact test was used for testing the difference between observed and expected proportions in mixes of viral isolates or between the inoculum and the supernatant in competition assays.”

Every comparison between proportions has been retested using Fisher’s exact test and the results section has been modified accordingly.

3) Specify important statisitcal parameters. For example, the statistical significant level was set at 0.05 (i.e. p < 0.05)

We have specified Line 302 “A p value < 0.05 was considered significant”

4) Specify statistical software used in this study including version and company.

We have specified Line 303 “Statistical analyses were performed using the GraphPad Prism Software 9.”

Results
-Khi² test (Line 260 and 263) and Chi² tests (Line 279, 285 and 288) should be changed to chi-squared tests or chi-square.

Thank you for this remark. Every comparison between proportions has been retested using Fisher’s exact test and the results section has been modified accordingly.

-Please report raw p-values in three decimal places (e.g. 0.001 - 0.009). The p-values could be reported as p<0.001 if they are smaller than 0.001.

We have now reported every p value <0.001 as such.

-Since the authors propose a part this work as a quantification assay, the precision of the quantification must be presented. The coefficient of variation in a percentage form (%CV) is usually used for precision and repeatability evaluation of quantitative PCR assays. Please calculate %CV for all quantitation assays presented in this study.

We have calculated the coefficient of variation across 14 independent quantitation assays performed with the same DENV-1, DENV-2 and DENV-3 standards.

We have given the result in the Results section Line 359-361:

“The coefficient of variation calculated for 5.08x10^3^ copies/µL of DENV-1, 5.16x10^3^ copies/µL of DENV-2 and 7.14x10^3^ copies/µL of DENV-3 across 14 independent experiments was 6.34%, 2.17% and 2.29% respectively.”

-Line 245 - 247: This assay allowed to determine the sensitivity of this serotype-specific qRT-PCR, which was higher than 5.08x101, 5.16x101, 7.14x101 and 1.36x101 genome copies/µL for DENV-1, -2, -3 and -4 respectively.
What are these reported numbers? The lower limit of detection (LLOD) or the lower limit of quantification (LLOQ)? Please describe how these numbers were derived in the materials and methods. One way to calculate LLOD is to perform PCR with serially diluted samples with known concentrations in replicates (preferably >= 9 replicates). Then calculate a probability of detection curve by logistic regression. Finally, calculate a concentration (for dilution) that has a probability of detection = 95%. For LLOQ, %CV must be derived first. Then acceptable %CV must be specified (e.g. %CV <= 35%). Finally, the lowest concentration with %CV < acceptable %CV must be calculated for LLOQ. (LLOQ is the lowest concentration that could be repeatedly measured with an acceptable range of variations or errors).

We have repeated the limit dilution experiment by performing ten-fold dilutions in duplicates for the DENV-1, -2, -3 and -4 reference RNAs from 7.52x10^5^ to 7.52x10^-2^ copies/µL, 1.19 x10^6^ to 1.19x10^-1^ copies/µL, 3.48x10^5^ to 3.48x10^-2^ copies/µL and 1.36x10^5^ to 1.36x10^-2^ copies/µL , respectively. We diluted the RNAs below 10^0^ copies/µL to reach signal extinction. Although we did not perform ≥9 replicates, to allow for the calculation of the LLOD, we were able to detect DENV-1 to DENV-4 RNA down to 7.52, 1.19, 3.48 and 1.36 copies/µL, respectively. We were also able to determine the linear range of the assay. We specified in the text:

Line 364-372:

Using these four RNA extracts of known concentration, a standard curve was established for each DENV-1 to -4 serotypes (Supplementary figure 4) as a serial ten-fold dilution of each RNAs performed in duplicate, allowing the absolute quantification of RNA extracts from DENV-1 to -4 with the multiplex serotype-specific qRT-PCR. This assay allowed to determine that the detection limit of this serotype-specific qRT-PCR was below 7.52, 1.19, 3.48 and 1.36 genome copies/µL for DENV-1, -2, -3 and -4 respectively (Supplementary figure 4). The linear range of the assay extended from 7.52x10^5^ to 7.52 copies/μL for DENV-1, 1.19x10^6^ to 1.19x10^1^ copies/µL for DENV−2, 3.48x10^5^ to 3.48x10^1^ copies/µL for DENV−3 and 1.36x10^5^ to 1.36x10^0^ copies/µL for DENV-4.

And in the abstract Line 25-26

“The qRT-PCR was specific, and had a limit of detection below 7.52, 1.19, 3.48 and 1.36 genome copies/µL.”

- Comparison of the replicative fitness of two DENV serotypes and Figure 4
While the authors proposed this work for a quantification assay, the main results were reported as proportions (%), not absolute quantification results. The use of these assays might be limited since there already are several serotype-specific or pan-serotype quantification assays that report the quantification results in absolute forms (i.e. copies/ml or log10-copies/ml). In addtion, digital RT-PCR could directly report an absolute count of dengue genomes without standard curves.

We indeed presented results as proportions of genome copy numbers of each strain in the inoculum and the supernatant. These proportions were derived from absolute counts. We provide an updated figure with absolute counts. We thank the reviewer for pointing out the existence of other quantitative serotype-specific assays, which we now report in the discussion section. However, these assays might fail to detect some strains, and especially DENV-3 sub-lineages. One of the strengths of our assay is the adaptation of primers and probes sequences to DENV-3 strains that have recently circulated in the Pacific region, where the number of sequenced DENV strains is low despite the importance of identifying the circulating serotype in this non-endemic zone where DENV serotype replacements have important epidemiological implications.

The quantification of dengue virus with qRT-PCR could have a variation up to +/- 1 log10-copies so any difference < 1 log10-copies might just be an error of the quantification. It would be interesting to see the variation (or %CV) of the quantification assays proposed here.

As detailed above, we have appended the %CV to the manuscript results section.

Figure 4C is the only panel in Figure 4 that show results as absolute counts. However, the scale of the y-axis is misleading as the viral load has a skewed distribution. The y-axis should be changed to a log10-scale.

We thank the reviewer for this suggestion. We have changed the y-axis to log10 scale.

Also were experiments for comparing the replicative fitness performed in replicates? If so, please specify the number of replicates in the materials and methods. If there were no replicates, how are the error bars in Figure 4D and 4E derived?

Experiments were performed in three independent replicates. We specified it in the figure legend and in the methods Line 293-294 “Experiments were performed in three independent replicates.”.

Discussion
- Line 302-306: Of importance, this optimized multiplex serotype-specific qRT-PCR may be used in diagnostic laboratories to identify the infecting DENV serotype and could easily be deployed on the field using field-compatible real-time thermocyclers, albeit possibly requiring fluorochrome adjustments.
1) There is no need for serotyping in clinical settings. The doctors do not need to know serotype. Dengue-infected patients confirmed by PCR will be treated the same regardless of serotype.
2) There is no need for dengue quantification in epidemiological study. Serotyping results might be useful for surveillance as the replacement of the current predominant serotype may indicate the new outbreak cycle.
3) The quantification results are generally and only used in research.
4) The point-of-care PCR that could identify the serotype of dengue virus plus detecting other arboviruses (e.g. Zika virus, Chikungunya virus) is now commercially available for clinical use ([https://www.sdbiosensor.com/product/product_view?product_no=23001](https://urldefense.com/v3/__https:/www.sdbiosensor.com/product/product_view?product_no=23001__;!!JFdNOqOXpB6UZW0!p-JK5mybjXP_f1Ti0Q_4ScaQRbLm_qE4_Hoje1QPG0DsiVcHhssbfL3ajJgM2fASvGH5fBClBuyfl2Md5Q$)).
We thank the reviewer for this invitation to modulate the significance of our results for clinicians. We have reformulated and deepened the discussion accordingly.

Line 491-499:

In recent years, understanding the role of genotypes and clades in DENV serotype replacements, as well as genotype- and clade-specific immunity, has become increasingly crucial. Serotyping dengue viruses thus plays a key role in the epidemiological study of the disease [50, 51].

Additionally, specific serotypes may be linked to different epidemics. Many studies have highlighted the importance of identifying events of DENV genotype and clade replacement, as seen, in the Americas [52], in Asia [53] and in the Pacific region [41]. In Brazil, the Ministry of Health therefore recommends that all suspected dengue samples be serotyped by PCR, enabling the evaluation of circulating serotypes [51].

Conclusion
Line 345-347: Such in vitro assessment of viral fitness may allow to evaluate the contribution of DENV replicative fitness to DENV serotype replacements in dengue-endemic or endemo-epidemic settings.

- Again, serotype replacements generally caused by host immune response and herd immunity. Please provide evidence that replicative fitness causes serotype replacements in real-life.
Recent publications evidence that replicative fitness modulates dengue epidemiology, favoring the emergence of new DENV variants.

We have added this information to the Discussion section Line 514-520:

For instance, a larger DENV-2 outbreak in Taiwan was associated with viral evolution since the previous outbreak. Acquisition of the NS5V357E mutation resulted in enhanced replicative fitness *in vitro* in mosquito and mammalian cells [6], highlighting the importance to assess viral replicative fitness. Similarly, emergence of the Asian I genotype of DENV-2 in Viet-Nam was associated with enhanced *in vivo* fitness denoted by higher viraemia in patients [54]. Furthermore, enhanced replicative fitness in the mosquito vector was observed in New Caledonia upon a genotype replacement of DENV-1 strains [48].Figures
- There are no descriptions or annotations of all figures.

We apologize for the absence of figure legends, which we now have appended at the end of the manuscript.
