# Supplementary material for: Development of a competition assay to assess the in vitro fitness of dengue virus serotypes using an optimized serotype-specific qRT-PCR
Source: PLoS One. 2025 Dec 15;20(12):e0339033. doi: 10.1371/journal.pone.0339033 (PMC12704846; doi:10.1371/journal.pone.0339033)
Supplement: Attachment — Submitted filename: PLOS_One_Inizan_2ndReview.docx [file pone.0339033.s011.docx]

Overall:

The authors have addressed the technical concerns and revised the manuscript such that the methodological aspects now meet the standards of PLOS ONE. Nevertheless, I remain unconvinced of the broader applicability of the reported assay. Its utility appears to be restricted to laboratory-controlled co-infection experiments in cell culture, which represent highly artificial conditions. As such, the assay is unlikely to have meaningful relevance to serotype replacement or other processes of epidemiological importance in natural settings. To strengthen the manuscript, the authors may consider clarifying the potential practical applications of this assay, while avoiding references to serotype replacement caused by differences in viral replication levels. For example, could it be used to titrate each serotype in a live attenuated dengue vaccine so that the vaccine will induce balanced immune response against all four serotypes? (Again, antigenicity of each serotype is also important for balancing immune response.) The authors may focus more on co-infection study. For example, Quintero-Gil et al. (<https://doi.org/10.3855/jidc.3978>) evaluated replicative fitness of two DENV serotypes infecting a mosquito cell line and a live mosquito.

There are several studies reporting that each serotype of DENV intrinsically replicates at different rates. Tricou et al. (<https://doi.org/10.1371/journal.pntd.0001309>), Douyen at al. (https://doi.org/10.1093/infdis/jir014), and Voung et al. (<https://doi.org/10.7554/eLife.92606.3>) reported that DENV-1 shows higher early/febrile-phase viral loads than DENV-2 and DENV-3 in dengue patients. However, serotype replacement still occurs among all four serotypes. Therefore, the ability of viral replication may have limited relevance, or even be irrelevant, to the serotype replacement observed in epidemiological surveillance.

The authors try to convince that the difference in replicative fitness play a very important role in serotype replacement. However, the citations included actually demonstrated replication fitness changes that might contribute to “genotype”, “lineage” and “clade” replacement, not serotype replacement. Genotype, lineage and clade are more difficult to identify requiring virus genome sequencing or specialized PCR. The assay proposed by the authors cannot identify genotype of DENV or evaluate replicative fitness of genotypes within the same serotype in a co-infection. (The authors mention this in Line 544-547).

*REF 6: Ko, H.-Y., et al., Emergence and increased epidemic potential of dengue variants with the NS5V357E mutation after consecutive years of transmission. iScience, 2024. 27(11): p. 110899. 🡪* The article reported an investigation of clade/strain replacement of DENV-2, not the serotype replacement.

*REF 35: Inizan, C., et al., Viral evolution sustains a dengue outbreak of enhanced severity. Emerg Microbes Infect, 2021. 10(1): p. 536-544. 🡪* The article focuses on DENV-1 evolution (i.e., genotype or strain replacement), not serotype replacements.

*REF 47: Gomgnimbou, M.K., et al., Utilization of novel molecular multiplex methods for the detection and, epidemiological surveillance of dengue virus serotypes and chikungunya virus in Burkina Faso, West Africa. Molecular Biology Reports, 2024. 51(1): p. 906.* 🡪 The article does not address replicative fitness or provide any information regarding virus load quantification.

*REF 48: O'Connor, O., et al., Potential role of vector-mediated natural selection in dengue virus genotype/lineage replacements in two epidemiologically contrasted settings. Emerg Microbes Infect, 2021. 10(1): p. 1346-1357.* 🡪 The article investigated genotype/lineage replacements within a serotype, not serotype replacement.

*REF 54: Vu, T.T., et al., Emergence of the Asian 1 genotype of dengue virus serotype 2 in Vietnam: in vivo fitness advantage and lineage replacement in South-East Asia. PLoS Negl Trop Dis, 2010. 4(7): p. e757 🡪* The article investigated lineage replacement within DENV-2, not serotype replacement.

Abstract:

Line 27 – 29: The qRT-PCR was specific, and had a limit of detection below 7.52, 1.19, 3.48 and 1.36 genome copies/µL, an efficiency of 1.993, 1.975, 1.902, 1.898 and a linearity (R²) of **0.99975, 0.99975,0.9985, 0.99965** for DENV-1, -2, -3 and -4 respectively.

Please report R² values with the same/consistent decimal places.

Discussion:

Line 451- 453: This serotype-specific qRT-PCR offers rapid, sensitive, and specific detection of DENV, allowing serotype differentiation and viral load quantification, which are crucial for efficient epidemiological surveillance [39-41].

The authors state that both serotype differentiation and viral load quantification are essential for effective epidemiological surveillance. While serotype differentiation is indeed widely recognized and applied in epidemiological analyses, viral load quantification is not typically used for this purpose. Of the three references cited, all specifically demonstrate the application of serotype data in epidemiological studies, but none provide evidence for the use of viral load quantification in this context. Consequently, the statement in lines 451–453 is not entirely accurate. To resolve this, either “viral load quantification” in the sentence should be removed, or an additional citation must be provided that clearly demonstrates its role in epidemiological surveillance.

*REF 39: Singh, K., et al., Identification of Dengue virus serotype and genotype: A comprehensive study from AIIMS Patna, Bihar. Indian Journal of Medical Microbiology, 2025. 53: p. 100789.* 🡪 Serotyping was done, but viral load quantification was not done. This reference does not support that “viral load quantification” is crucial for epidemiological surveillance.

*REF 40: Dieng, I., et al., Multifoci and multiserotypes circulation of dengue virus in Senegal between 2017 and 2018. BMC Infectious Diseases, 2021. 21(1): p. 867.* 🡪 Serotyping was done, but viral load quantification was not done. This reference does not support that “viral load quantification” is crucial for epidemiological surveillance.

*REF 41: Dupont-Rouzeyrol, M., et al., Epidemiological and molecular features of dengue virus type-1 in New Caledonia, South Pacific, 2001–2013. Virology Journal, 2014. 11(1): p.61.* 🡪 Serotyping was done, but viral load quantification was not done. This reference does not support that “viral load quantification” is crucial for epidemiological surveillance.

Line 512 – 514: To improve dengue outbreak prevention and the surveillance of antiviral resistance [46], it is crucial to strengthen genomic surveillance but also to better assess viral replicative fitness [47].

*REF 47: Gomgnimbou, M.K., et al., Utilization of novel molecular multiplex methods for the detection and, epidemiological surveillance of dengue virus serotypes and chikungunya virus in Burkina Faso, West Africa. Molecular Biology Reports, 2024. 51(1): p. 906.* 🡪 The article does not address replicative fitness or provide any information regarding virus load quantification.
