# Supplementary material for: Development of a competition assay to assess the in vitro fitness of dengue virus serotypes using an optimized serotype-specific qRT-PCR
Source: PLoS One. 2025 Dec 15;20(12):e0339033. doi: 10.1371/journal.pone.0339033 (PMC12704846; doi:10.1371/journal.pone.0339033)
Supplement: Attachment — Submitted filename: Response to Reviewers2nd round20251113.docx [file pone.0339033.s012.docx]

Please find below a point-by-point response to reviewers’ comments.

Reviewer #1: The revised manuscript by Griffon and colleagues entitled “Development of a competition assay to assess the in vitro fitness of dengue virus serotypes using an optimized serotype-specific qRT-PCR” is much improved compared to the previous version. The authors’ efforts in revising the manuscript are commendable, and their responses to the queries are satisfactory.

Few things to consider…

Critical comments:
Why was the envelope (E) gene chosen for serotype-specific RT-PCR in DENV infection? Please provide a brief explanation in the Introduction section or a detailed explanation in the discussion section.

Thank you for this suggestion. We have added to the introduction section, lines 81-86:

Historically, dengue phylogenies mostly relied on E gene sequences [23]. As the E protein is a primary target for host immune pressure, it accumulates phylogenetically informative variations. Furthermore, E sequence variation correlates with serotype/genotype structure, making it useful for inferring lineage/strain definitions. Therefore, several reliable DENV serotyping assays are based on E gene sequences [22, 24].

Specific comments:

Line 109-115: Please include the justification for using HuH7 cell line in this paragraph. This will help in understanding why HuH7 cells were used in the study.

We specified in the manuscript lines 119-123:

In humans, DENV mostly replicates in hepatocytes and cells from the monocytic lineage [25]. We therefore used the human hepatocarcinoma HuH7 cell line, which was cultured at 37°C under 5% CO_2_ in Dulbecco's Modified Eagle Medium (DMEM, Gibco™, Fisher scientific, Paisley, UK) supplemented with 10% decomplemented FCS.

Line 374-376: //… a DENV-1/DENV-2, a DENV-1/DENV-4, a DENV-2/DENV-3, a DENV-2/DENV-4 and a DENV-3/DENV-4//. Please replace ‘a’ with ‘1’.

We have modified lines 381-384:

Eighty-seven serum samples from DENV patients were included in a specificity/sensitivity assay, including 32 DENV-1, 20 DENV-2, 13 DENV-3, 7 DENV-4, one DENV-1/DENV-2, one DENV-1/DENV-4, one DENV-2/DENV-3, one DENV-2/DENV-4 and one DENV-3/DENV-4 co-infection and 10 negative samples according to the routine technique [34].

Line 383-386: //Regarding the co-infections, while the routine technique detected two co-infections (DENV-2/DENV-3 and DENV-2/DENV-4) out the five co-infections historically determined at the time of sample collection, the current optimized qRT-PCR detected three co-infections (DENV-1/DENV-4, DENV-2/DENV-3, DENV-3/DENV-4)//. Since you describe the co-infections as ‘historically determined,’ does this mean they were previously reported by other researchers, or are they based on your current observations? Please clarify and include this information.

These co-infections were “historically” determined by the diagnosis laboratory at the time of diagnosis. Probably due to sample degradation, the routine qRT-PCR historically used at the time of diagnosis and still in use by the diagnosis laboratory was not able to confirm these co-infections when recently run on the same samples preserved at -80°C.

We specified lines 390-395:

Co-infections were both reassessed by the routine serotyping technique historically used at the time of diagnosis and assessed by the current optimized qRT-PCR. While the routine technique detected two co-infections (DENV-2/DENV-3 and DENV-2/DENV-4) out the five co-infections historically determined by the same technique at the time of sample collection, the current optimized qRT-PCR detected three co-infections (DENV-1/DENV-4, DENV-2/DENV-3, DENV-3/DENV-4).

And added lines 405-410:

Of note, the Ct for mono-infections did not notably increase with the duration since sample retrieval, indicating low viral RNA degradation in our collection of serum samples. However, three co-infections could not be confirmed by the routine technique and two could not be confirmed by the current optimized qRT-PCR, nonetheless suggesting a partial RNA degradation impairing the appropriate detection of some co-infections.

Line 394-395: // Only one sample typed as DENV-2 by the routine technique was identified as DENV-3 positive by our technique, yielding one false positive//. Do you think this discrepancy could be due to sequencing of a specific gene rather than the complete genome? Discuss…

In the current manuscript, we describe the development of a qRT-PCR. Sequencing is indeed the method of choice to accurately confirm serotype assignment. Sequencing of the DENV strain contained in this serum sample could indeed confirm the actual infecting serotype. It is possible that due to sequence similarity, the current optimized serotype-specific PCR or the routine technique mistakenly assigned the wrong serotype to this putative atypical sequence. We added lines 404-405:

Whole-genome sequencing of this strain could identify the correct assignment.

Figure 4: Please provide color legend within the figure for D, E, F, & G.

We apologize for this mistake. We have added lines 655-662:

**D-E.** Absolute concentration of each isolate in the inoculum (**D.**) and the supernatant (**E.**) of HuH7 cells infected with a viral suspension containing a mix of DENV-1:DENV-2, DENV-1:DENV-3 or DENV-3:DENV-2 at a ratio 50:50. **F-G.** Absolute concentration of each isolate in the inoculum (**F.**) and the supernatant (**G.**) of HuH7 cells infected with a viral suspension containing a mix of DENV-1:DENV-2, DENV-1:DENV-3 or DENV-3:DENV-2 at a ratio 10:90. The concentration was determined using the quantitative serotype-specific multiplex RT-PCR. Mean and Standard Deviations of three independent triplicates are shown in **D-G**.

Reviewer #2: Thank you very much for your great improvement of your manuscript and satisfactory revision. No more revision required.

We thank the Reviewer for this positive appraisal.

Reviewer #3: I have no more questions, it seems that the authors do a good job and all my concerns have been addressed.

We thank the Reviewer for this positive feed-back.

Reviewer #4: For a formatted version please see the attached file.

Overall:
The authors have addressed the technical concerns and revised the manuscript such that the methodological aspects now meet the standards of PLOS ONE. Nevertheless, I remain unconvinced of the broader applicability of the reported assay. Its utility appears to be restricted to laboratory-controlled co-infection experiments in cell culture, which represent highly artificial conditions. As such, the assay is unlikely to have meaningful relevance to serotype replacement or other processes of epidemiological importance in natural settings. To strengthen the manuscript, the authors may consider clarifying the potential practical applications of this assay, while avoiding references to serotype replacement caused by differences in viral replication levels. For example, could it be used to titrate each serotype in a live attenuated dengue vaccine so that the vaccine will induce balanced immune response against all four serotypes? (Again, antigenicity of each serotype is also important for balancing immune response.)

We thank the reviewer for this interesting and innovative suggestion. We have added to the discussion section lines 484-487:

Serotyping dengue viruses could be used in vaccine development: the current optimized serotype-specific qRT-PCR could allow the relative quantification of vaccine strains from each of the four serotypes in a live attenuated vaccine, allowing to balance the inoculum and elicit an effective pan-serotype immunity.

The authors may focus more on co-infection study. For example, Quintero-Gil et al. ([https://doi.org/10.3855/jidc.3978](https://urldefense.com/v3/__https:/doi.org/10.3855/jidc.3978__;!!JFdNOqOXpB6UZW0!o8uWPIx4CFMCAzvzrYZBR3e2r0uf9z1_70mfNRw7L1kZMx9bJ4mbh4Rk3I0u7UD349ro0sYYJSdif9B67g$)) evaluated replicative fitness of two DENV serotypes infecting a mosquito cell line and a live mosquito.

We thank the Reviewer for this identification of another study analyzing DENV serotypes replicative fitness in competition assay. We have added this reference in the existing relevant paragraph lines 73-76:

Competition in mosquito C6/36 cells or adult mosquitoes between two DENV serotypes were also reported; they were probed using type-specific hyper-immune mouse ascites [17, 18], monoclonal antibodies [18] or a reverse transcription followed by a SybR Green qPCR using serotype-specific primers [19, 20].

And to the discussion section lines 526-527:

For DENV as well, competition assays allow to specifically detect significant differences in fitness between two different strains, belonging to two different serotypes for instance [17-20].

There are several studies reporting that each serotype of DENV intrinsically replicates at different rates. Tricou et al. ([https://doi.org/10.1371/journal.pntd.0001309](https://urldefense.com/v3/__https:/doi.org/10.1371/journal.pntd.0001309__;!!JFdNOqOXpB6UZW0!o8uWPIx4CFMCAzvzrYZBR3e2r0uf9z1_70mfNRw7L1kZMx9bJ4mbh4Rk3I0u7UD349ro0sYYJSeIc_LPkw$)), Douyen at al. ([https://doi.org/10.1093/infdis/jir014](https://urldefense.com/v3/__https:/doi.org/10.1093/infdis/jir014__;!!JFdNOqOXpB6UZW0!o8uWPIx4CFMCAzvzrYZBR3e2r0uf9z1_70mfNRw7L1kZMx9bJ4mbh4Rk3I0u7UD349ro0sYYJSfwFOm8aw$)), and Voung et al. ([https://doi.org/10.7554/eLife.92606.3](https://urldefense.com/v3/__https:/doi.org/10.7554/eLife.92606.3__;!!JFdNOqOXpB6UZW0!o8uWPIx4CFMCAzvzrYZBR3e2r0uf9z1_70mfNRw7L1kZMx9bJ4mbh4Rk3I0u7UD349ro0sYYJSfw6MhWDA$)) reported that DENV-1 shows higher early/febrile-phase viral loads than DENV-2 and DENV-3 in dengue patients. However, serotype replacement still occurs among all four serotypes. Therefore, the ability of viral replication may have limited relevance, or even be irrelevant, to the serotype replacement observed in epidemiological surveillance.

We thank the reviewer for this insightful comment. We have added lines 502-505:

Simultaneously, several studies report differences in viral load in patients according to the infecting DENV serotype [56-58], suggesting differences in replicative fitness between DENV serotypes. Their contribution to DENV serotype replacements remains elusive.

The authors try to convince that the difference in replicative fitness play a very important role in serotype replacement. However, the citations included actually demonstrated replication fitness changes that might contribute to “genotype”, “lineage” and “clade” replacement, not serotype replacement. Genotype, lineage and clade are more difficult to identify requiring virus genome sequencing or specialized PCR. The assay proposed by the authors cannot identify genotype of DENV or evaluate replicative fitness of genotypes within the same serotype in a co-infection. (The authors mention this in Line 544-547).
REF 6: Ko, H.-Y., et al., Emergence and increased epidemic potential of dengue variants with the NS5V357E mutation after consecutive years of transmission. iScience, 2024. 27(11): p. 110899. ◊ The article reported an investigation of clade/strain replacement of DENV-2, not the serotype replacement.
REF 35: Inizan, C., et al., Viral evolution sustains a dengue outbreak of enhanced severity. Emerg Microbes Infect, 2021. 10(1): p. 536-544. ◊ The article focuses on DENV-1 evolution (i.e., genotype or strain replacement), not serotype replacements.
REF 47: Gomgnimbou, M.K., et al., Utilization of novel molecular multiplex methods for the detection and, epidemiological surveillance of dengue virus serotypes and chikungunya virus in Burkina Faso, West Africa. Molecular Biology Reports, 2024. 51(1): p. 906. ◊ The article does not address replicative fitness or provide any information regarding virus load quantification.
REF 48: O'Connor, O., et al., Potential role of vector-mediated natural selection in dengue virus genotype/lineage replacements in two epidemiologically contrasted settings. Emerg Microbes Infect, 2021. 10(1): p. 1346-1357. ◊ The article investigated genotype/lineage replacements within a serotype, not serotype replacement.
REF 54: Vu, T.T., et al., Emergence of the Asian 1 genotype of dengue virus serotype 2 in Vietnam: in vivo fitness advantage and lineage replacement in South-East Asia. PLoS Negl Trop Dis, 2010. 4(7): p. e757 ◊ The article investigated lineage replacement within DENV-2, not serotype replacement.

We agree with the reviewer that we should clarify the fact that the studies we refer to considered genotype/lineage/clade replacements and not serotype replacements. We have specified lines 498-505:

However, emergence or maintenance of DENV serotypes in some regions despite high herd immunity [39] and an increasing number of studies [6, 55, 56] suggest the contribution of virological factors in the epidemiological fitness of a given DENV strain belonging to an emerging genotype, lineage or clade, including its replicative fitness. Simultaneously, several studies report differences in viral load in patients according to the infecting DENV serotype [57-59], suggesting differences in replicative fitness between DENV serotypes. Their contribution to DENV serotype replacements remains elusive.

We have also deleted lines 482-483 in the discussion section:

In recent years, understanding the role of genotypes and clades in DENV serotype replacements, as well as genotype- and clade-specific immunity, has become increasingly crucial.

Abstract:
Line 27 – 29: The qRT-PCR was specific, and had a limit of detection below 7.52, 1.19, 3.48 and 1.36 genome copies/µL, an efficiency of 1.993, 1.975, 1.902, 1.898 and a linearity (R²) of 0.99975, 0.99975,0.9985, 0.99965 for DENV-1, -2, -3 and -4 respectively.
Please report R² values with the same/consistent decimal places.

We apologize for this mistake and have corrected the R² for DENV-3 to 0.99850, both in the abstract and line 371.

Discussion:
Line 451- 453: This serotype-specific qRT-PCR offers rapid, sensitive, and specific detection of DENV, allowing serotype differentiation and viral load quantification, which are crucial for efficient epidemiological surveillance [39-41].
The authors state that both serotype differentiation and viral load quantification are essential for effective epidemiological surveillance. While serotype differentiation is indeed widely recognized and applied in epidemiological analyses, viral load quantification is not typically used for this purpose. Of the three references cited, all specifically demonstrate the application of serotype data in epidemiological studies, but none provide evidence for the use of viral load quantification in this context. Consequently, the statement in lines 451–453 is not entirely accurate. To resolve this, either “viral load quantification” in the sentence should be removed, or an additional citation must be provided that clearly demonstrates its role in epidemiological surveillance.

REF 39: Singh, K., et al., Identification of Dengue virus serotype and genotype: A comprehensive study from AIIMS Patna, Bihar. Indian Journal of Medical Microbiology, 2025. 53: p. 100789. ◊ Serotyping was done, but viral load quantification was not done. This reference does not support that “viral load quantification” is crucial for epidemiological surveillance.
REF 40: Dieng, I., et al., Multifoci and multiserotypes circulation of dengue virus in Senegal between 2017 and 2018. BMC Infectious Diseases, 2021. 21(1): p. 867. ◊ Serotyping was done, but viral load quantification was not done. This reference does not support that “viral load quantification” is crucial for epidemiological surveillance.
REF 41: Dupont-Rouzeyrol, M., et al., Epidemiological and molecular features of dengue virus type-1 in New Caledonia, South Pacific, 2001–2013. Virology Journal, 2014. 11(1): p.61. ◊ Serotyping was done, but viral load quantification was not done. This reference does not support that “viral load quantification” is crucial for epidemiological surveillance.

We thank the reviewer for this suggestion. We have modified lines 464-466 as follows:

This serotype-specific qRT-PCR offers rapid, sensitive, and specific detection of DENV, allowing serotype differentiation and viral load quantification. Serotype differentiation is crucial for efficient epidemiological surveillance [42-44].

Line 512 – 514: To improve dengue outbreak prevention and the surveillance of antiviral resistance [46], it is crucial to strengthen genomic surveillance but also to better assess viral replicative fitness [47].
REF 47: Gomgnimbou, M.K., et al., Utilization of novel molecular multiplex methods for the detection and, epidemiological surveillance of dengue virus serotypes and chikungunya virus in Burkina Faso, West Africa. Molecular Biology Reports, 2024. 51(1): p. 906. ◊ The article does not address replicative fitness or provide any information regarding virus load quantification.

We thank the reviewer for his/her carefully reading of this section. We moved the reference upstream in the sentence for accuracy, lines 506-508:

To improve dengue outbreak prevention but also the surveillance of antiviral resistance [60], it is nonetheless crucial to strengthen genomic surveillance [61] and better assess viral replicative fitness.
